# A novel device to assess the oxygen saturation and congestion status of the gastric conduit in thoracic esophagectomy

**DOI:** 10.1186/s12893-023-02303-0

**Published:** 2024-01-08

**Authors:** Takeo Fujita, Takashi Shigeno, Daisuke Kajiyama, Kazuma Sato, Naoto Fujiwara, Hiroyuki Daiko

**Affiliations:** Division of Esophageal Surgery, National Cancer Center Hospital East, 6-5-1 Kashiwanoha, Kashiwa, Chiba, 277-8577 Japan

**Keywords:** Esophagectomy, Gastric conduit, Oxygen saturation, Total hemoglobin index

## Abstract

**Background:**

In thoracic esophagectomy, anastomotic leakage is one of the most important surgical complications. Indocyanine green (ICG) is the most widely used method to assess tissue blood flow; however, this technique has been pointed out to have disadvantages such as difficulty in evaluating the degree of congestion, lack of objectivity in evaluating the degree of staining, and bias easily caused by ICG injection, camera distance, and other factors.

Evaluating tissue oxygen saturation (StO2) overcomes these disadvantages and can be performed easily and repeatedly. It is also possible to measure objective values including the degree of congestion. We evaluate novel imaging technology to assess tissue oxygen saturation (StO2) in the gastric conduit during thoracic esophagectomy.

**Methods:**

Fifty patients were enrolled, with seven excluded due to intraoperative findings, leaving 43 for analysis. These patients underwent thoracic esophagectomy for esophageal cancer. The device was used intraoperatively to evaluate tissue oxygen saturation (StO2) and total hemoglobin index (T-HbI), which guided the optimal site for gastric tube anastomosis. The efficacies of StO2 and T-HbI in relation to short-term outcomes were analyzed.

**Results:**

StO2, indicating blood supply to the gastric tube, remained stable beyond the right gastroepiploic artery (RGEA) end but significantly decreased distally to the demarcation line (*p* <  0.05). T-HbI, indicative of congestion, significantly decreased past the RGEA (*p* <  0.05). Three patients experienced anastomotic leakage. These patients exhibited significantly lower StO2 (*p* <  0.01) and higher T-HbI (*p* <  0.01) at both the RGEA end and the demarcation line. Furthermore, the anastomotic site, usually within 3 cm of the RGEA’s anorectal side, also showed significantly lower StO2 (*p* <  0.01) and higher T-HbI (*p* <  0.01) in patients with anastomotic leakage.

**Conclusions:**

The novel device provides real-time, objective evaluations of blood flow and congestion in the gastric tube. It proves useful for safer reconstruction during thoracic esophagectomy, particularly by identifying optimal anastomosis sites and predicting potential anastomotic leakage.

**Supplementary Information:**

The online version contains supplementary material available at 10.1186/s12893-023-02303-0.

## Background

Gastrointestinal anastomosis is of the utmost importance in gastrointestinal surgery, as anastomotic leakage prolongs the postoperative hospital stay and increases healthcare costs. Furthermore, anastomotic leakage can also negatively affect the long-term quality of life and prognosis [1, 2]. In thoracic esophagectomy, the risk of anastomotic leakage is reduced by properly assessing the blood flow to the reconstructed organ, namely the gastrointestinal tract, to determine the optimal anastomotic site. One method that is widely used to assess the blood flow to determine the optimal anastomotic site is indocyanine green (ICG) imaging [3, 4]. Recent clinical studies report that ICG imaging of the stomach reduces the incidence of anastomotic leakage [5, 6]. However, administration of ICG may cause allergic reactions. Furthermore, ICG imaging analysis of blood perfusion is qualitative, not quantitative.

A new device has recently been developed to assess blood perfusion in the tissue in real time by analyzing the tissue oxygen saturation (StO2) and congestion [7, 8]. This device quantitatively measures perfusion and stasis without the administration of fluorescent or contrast agents and can measure the entire gastrointestinal tract in real time during anastomosis procedures, suggesting that its clinical use may minimize the occurrence of anastomotic leakage. The purpose of this study was to evaluate the efficacy of this novel blood flow assessment device to assess gastrointestinal tract perfusion for anastomotic integrity in patients undergoing thoracic esophagectomy.

## Methods

### Study design

**Fig. 1:**
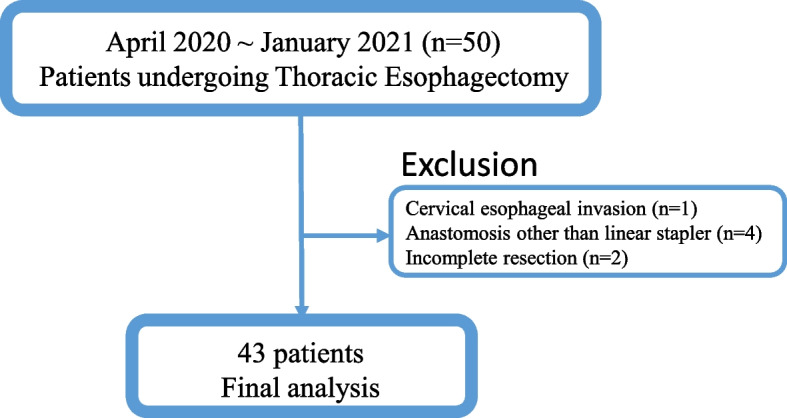
Flow chart of the patients’ recruitment. Between April 2020 to January 2021, fifty patients who met the eligibility criteria for radical thoracic esophageal cancer surgery were enrolled. Of these, one case with intraoperative evidence of cervical esophageal invasion, four cases in which safe anastomosis with linear staple was judged to be difficult based on surgical findings, and two cases in which the cancer was incompletely resected were excluded as ineligible. Forty-three patients were included in the final analysis

This single-center prospective cohort study was performed between April 2020 and January 2021 at the National Cancer Center Hospital East in Japan. The study flowchart is shown in Fig. 1. Patients undergoing thoracic esophagectomy for esophageal cancer who underwent intraoperative evaluation of the StO2 and total hemoglobin index (T-HbI) immediately after construction of the gastric conduit were analyzed. Participants were selected from 50 esophageal cancer patients who visited our esophageal cancer clinic and indicated their willingness to participate in the study. Informed consent was obtained by providing patients with a written description of the study’s purpose, protocol, and risks and benefits and all patients provided written informed consent.

The study period is 10 months, from January 2020 to April 2021. The study was approved by the Ethics Committee (approval number #2018–248).

Participants were esophageal cancer patients who had received a reconstructed stomach tube and had stomach tube blood flow assessment performed using the Toccare device. The device provides a noninvasive method for quantitative blood flow assessment.

For the evaluation, the device was used to quantitatively measure the participant’s stomach tube blood flow. Measurements were taken at specific anatomical points during surgery. The evaluation included analysis of blood flow parameters based on data obtained from the device.

Statistical methods and appropriate descriptive statistics were used to analyze the data. The primary endpoints were the efficacy, safety, and quantitative value of the Toccare device in assessing gastric tube blood flow. Secondary endpoints considered were postoperative complications and results related to stomach tube reconstruction.

Study results were processed according to the principles of anonymization and confidentiality and do not contain personally identifiable information. Training and quality control of data collection and analysis protocols were also implemented to minimize study limitations and bias.

### Patients

Fifty patients who underwent thoracic esophagectomy for esophageal cancer at the National Cancer Center Hospital East in Japan were investigated. Preoperative diagnoses were based on imaging studies, namely upper gastrointestinal studies, endoscopic examination, and conventional computed tomography. Histological evaluation of endoscopic biopsy specimens was performed for all patients. The preoperative tumor stage, histopathological findings, surgical procedures performed, and outcomes were recorded.

The inclusion criteria for this study were (1) aged over 20 years; (2) diagnosed with malignant thoracic esophageal cancer; (3) a pretreatment clinical disease stage of cT1-4aN0–3; (4) a European Cooperative Oncology Group performance status of 0–1; and (5) an assumption that reconstruction by stomach tube with cervical anastomosis using the mechanical linear stapling technique was possible during surgery. The exclusion criteria were (1) cervical and abdominal esophageal cancer; (2) any history of definitive chemoradiation treatment of the esophagus; (3) cT4b tumors; and (4) anastomosis via a method other than the mechanical linear stapling technique.

Written informed consent was obtained from all patients. The study was approved by the Committee for ethics of the National Cancer Center (Japan) (approval number #2018–332). Also, this study confirms to the provisions of the Declaration of Helsinki (as revised in Tokyo 2004).

### Novel device to evaluate the oxygen saturation and total hemoglobin index

**Fig. 2:**
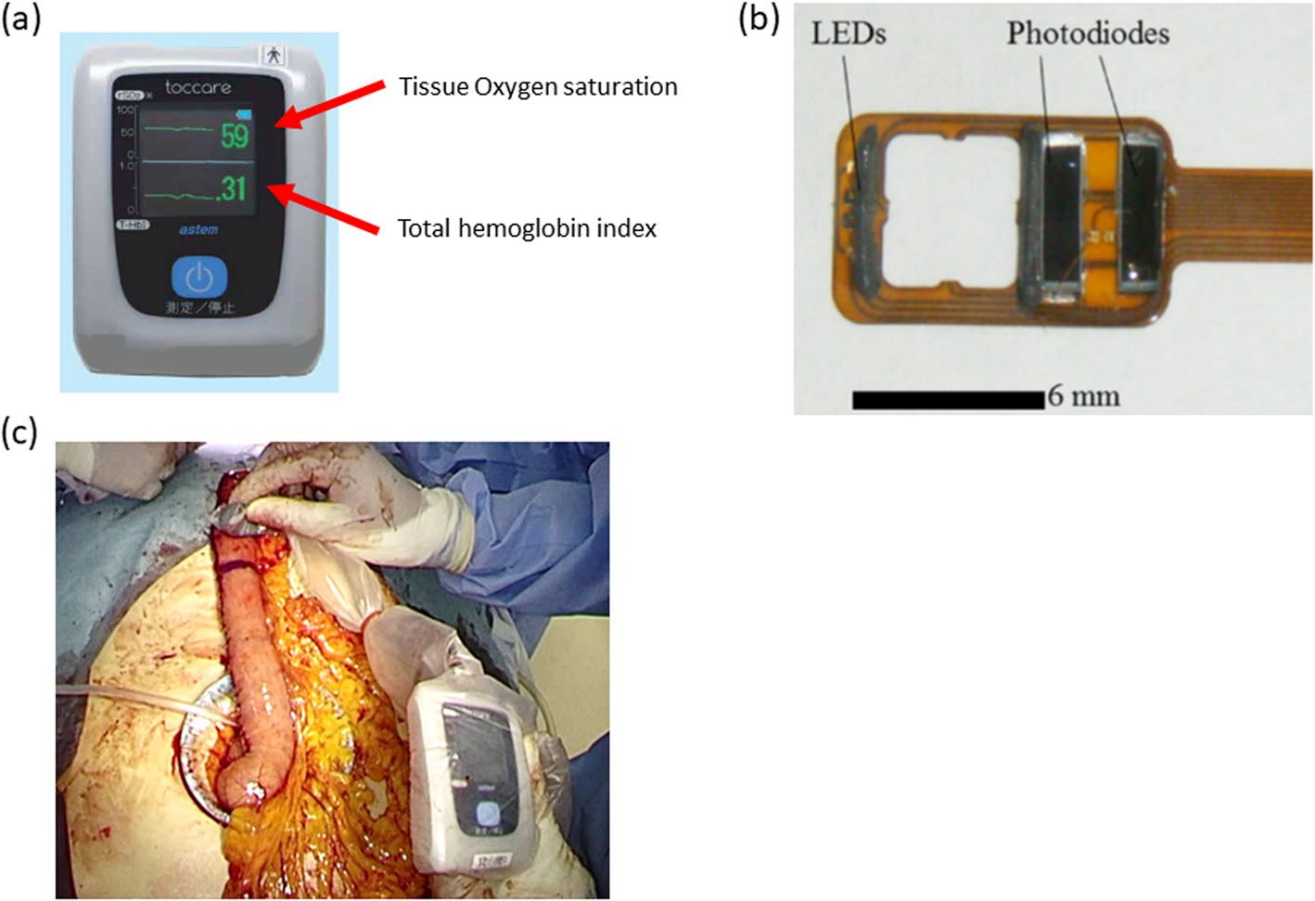
Overall view of the oxygen saturation and tissue congestion evaluation device. **a** Appearance of the display of this device. The upper panel displays tissue oxygen saturation, and the lower panel displays T-Hb index as a congestion index. By placing the detector in close contact with the target organ, these two parameters are immediately analyzed and displayed. These values can be measured repeatedly in real time without the need for reagents. **b** Overview of the internal structure of the device’s detector. Tissue oxygen saturation quantifies the value obtained by projecting the target tissue from two different photodiodes. **c** Representative images of an intraoperative real time evaluation for oxygen saturation & T-HbI of the gastric tube obtained during esophagectomy

**Fig. 3:**
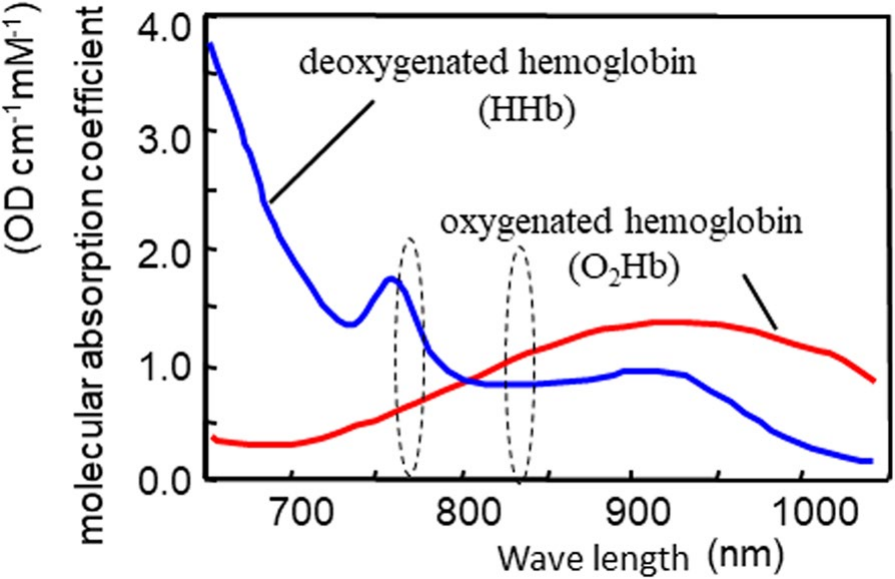
Signal of the wavelength of light to be analyzed and the characteristics of the device. This device can obtain images from the surface of the organ as well as the body surface, acquiring the signals from the processor unit. The unit calculates a StO_2_ value by using consecutive two different wavelength illuminations

The novel Toccare™ device (Astem Corporation, Kawasaki, Japan) was used to analyze the StO2 and T-HbI (Fig. 2). The Toccare™ provides the regional StO2 per unit of volume of targeted biological tissue. It also provides a T-HbI value that indicates regional tissue congestion levels by calculating the hemoglobin value per unit volume of tissue. Briefly, the Toccare™ consists of two LED lights and their receptors (Fig. 2b). The regional StO2 and T-HbI are displayed simultaneously on the separate monitors in real time (Fig. 2c). The Toccare™ derives the StO2 images from the differences in the absorption coefficient in the visible light region between oxy- and deoxyhemoglobin using a small number of wavelengths (Fig. 3). The details of this Toccare™ device are described elsewhere [9].

### Anastomotic procedure

In all cases, thoracic esophagectomy was performed under the direction of the regular attending surgeon. For transthoracic esophagectomy, subtotal resection of the esophagus was performed with three-field regional lymph node dissection, regardless of tumor stage [10]. For thoracoscopic esophagectomy, we preserved the azygos arch and the right bronchial artery [10]. The laparoscopic approach was principally used for the abdominal portion of the operation, except in patients with bulky lymph node metastases or a history of laparotomy [10]. The esophagus was usually reconstructed with a gastric tube via the retrosternal route [10].

The conduit was designed as a narrow gastric tube using the curved-shape stapler (Endo GIA™ Radial Reload with Tri-Staple™; Medtronic, Minneapolis, MN, USA) in the first stapling. The right gastric artery is ligated in all cases before the first stapling. A linear stapler (Endo GIA™ with Tri-Staple™; Medtronic) was then used. The anastomosis was performed using the modified Collard technique with a 45-mm linear stapler posteriorly and 45-mm and 60-mm linear staplers anteriorly. The detail of the procedure was described previously [10].

### Anesthesia and intraoperative management during thoracic esophagectomy

Routine monitoring was initiated upon arrival in the operating room, namely electrocardiography, noninvasive blood pressure monitoring, pulse oximetry, and capnography. Anesthesia was then induced with 1.5–2.5 mg/kg propofol, 1–2 μg/kg fentanyl, and 0.1 mg/kg vecuronium, and was maintained with 3% end-tidal sevoflurane in oxygen until tracheal intubation. After intubation, anesthesia was maintained with 2% end-tidal sevoflurane at 40% oxygen (air/oxygen mixture at 4 L/min) supplemented with doses of fentanyl and vecuronium.

After the operation, the endotracheal tube was removed. Once the patient achieved a modified Aldrete score of > 9, they were transported from the operating room to the intensive care unit (ICU), as described previously [10].

### Quantitative evaluation of regional tissue oxygen saturation and congestion

**Fig. 4:**
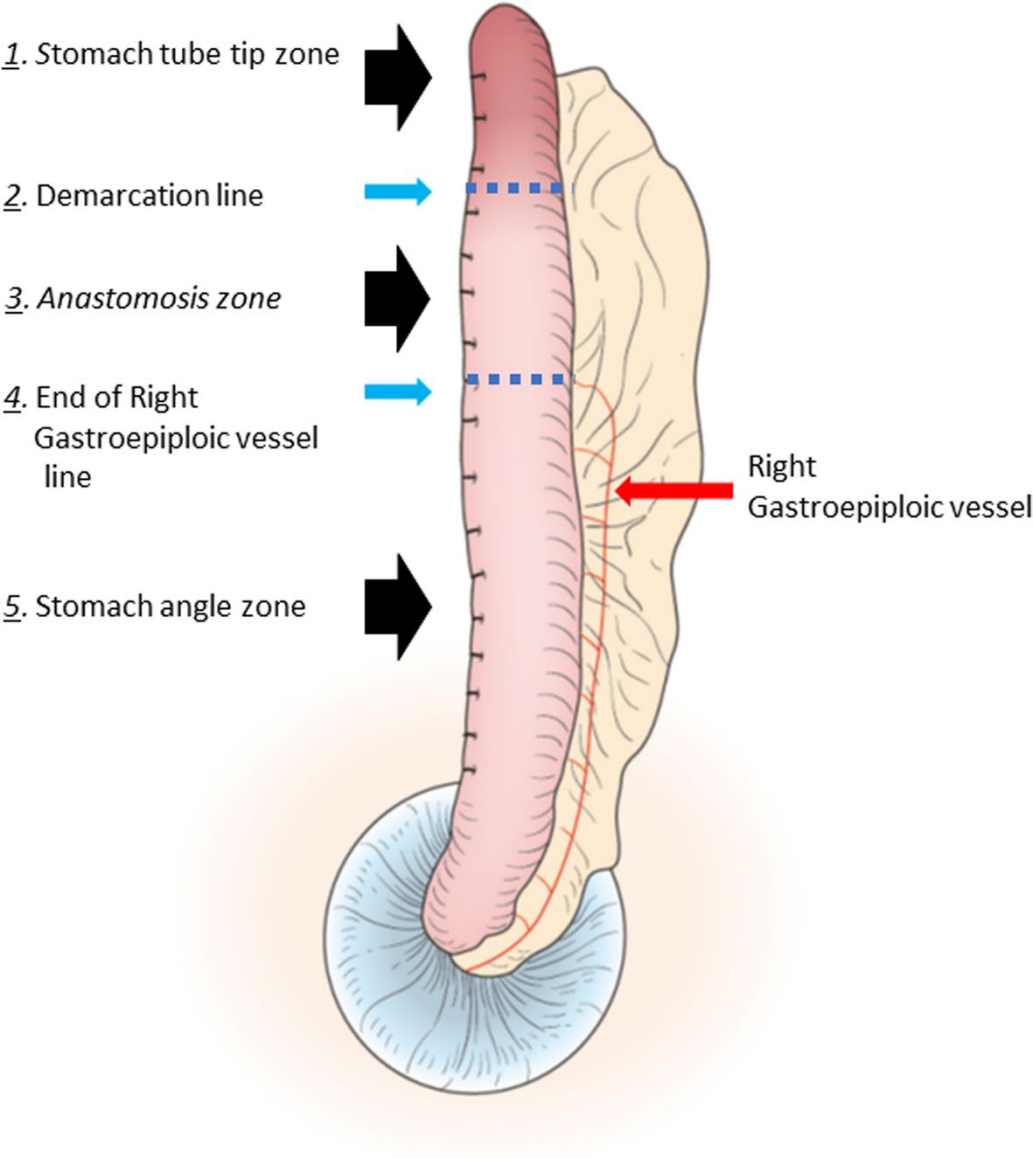
Schematic representation of the sites and the boundaries of stomach tube for tissue oxygen saturation assessment. Two important boundaries in the post-production gastrointestinal tract (demarcation line and end of right gastroepiploic vessel line) and three gastrointestinal tract regions (stomach angle zone, anastomosis zone, stomach tube tip zone) divided by the demarcation line. Stomach tube tip zone)

The evaluation procedures were recorded on video and the stored still images were used to quantitatively evaluate the StO2 and T-HbI using software developed by the Astem Corporation. The regions of interest were three areas on the gastric tube (stomach tube tip zone, anastomosis zone, stomach angle zone) and two reference lines (demarcation line, line at the end of the right gastroepiploic vessel). The stomach angle zone, the height where the gastric angle was located on the exact the point of the original stomach was marked on the greater curvature side, and that area was used as the measurement point. The anastomosis zone was measured at the anastomotic position of the temporally elevated stomach tube (in most cases, it was located between the end of the RGEA and the demarcation line). The stomach tube tip zone was literally the tip of the stomach tube. The StO2 and congestion (i.e., the T-HbI) were calculated for each region of interest (Fig. 4). In addition, the distance from the end of the right gastroepiploic vessel to the anastomotic site of the gastric tube was recorded and the StO2 of the region was calculated. The average StO2 and T-HbI of each patient were compared among the regions of interest.

### Definitions of surgical complications

Surgical complications were evaluated using the Clavien-Dindo classification system [11]. Complications classified as grade 2 and higher were defined as surgical complications. Surgical site infection was defined according to the Surgical Wound Infection Task Force 1 guidelines and included infections at the incision site or within the organs/spaces manipulated during surgery. Respiratory infection was defined as the presence of new or progressive infiltrates on chest radiography plus at least two of the following signs: temperature > 38 °C, purulent sputum, white blood cell count > 1 × 10^4^/mm^3^ or < 4 × 10^3^/mm^3^, and signs of inflammation on auscultation, as described previously [10].

### Perioperative management

The same postoperative clinical management pathway (CMP) was used for all patients, regardless of the type of abdominal approach. All patients received enteral nutrition through a nasal feeding tube until the start of oral intake on postoperative day (POD) 6. Briefly, fluid balance was achieved through a peripheral line, with additional enteral feeding on POD 1. Enteral nutrition was discontinued after the absence of anastomotic leakage was confirmed on POD 6.

Perioperative management was performed by the same clinical staff in the same ICU (POD 1 and 2) and ward (POD 3 and later). The endotracheal tube was removed from all patients in the operating room or immediately upon arrival in the ICU. On POD 6, a radiographic contrast agent swallow examination was performed to evaluate the anastomosis and any passage problems. If this examination showed no leakage or obstruction, the nasogastric tube was removed and oral intake was initiated in accordance with the CMP. In the absence of any complications, the patient was enrolled in the postoperative rehabilitation program and discharged on POD 12–20, as described previously [10].

Any abnormal clinical findings after surgery, such as hypoxia, leukocytosis, or abnormal pleural drainage, were investigated using computed tomography and/or other radiographic examinations to diagnose and optimally manage the abnormality as soon as possible, as described previously [10].

### Statistical analysis

Statistical analyses were performed using R software (R Foundation, Vienna, Austria). Intergroup differences were compared using the chi-squared test and the Mann-Whitney U-test. *P* < 0.05 was considered to indicate a significant difference.

## Results

A total of 50 consecutive patients were enrolled in the present study. After enrollment, seven patients were deemed ineligible and excluded because of intraoperative findings; one excluded patient had tumor invasion into the cervical esophagus discovered intraoperatively, four patients underwent anastomosis by other methods because the linear stapling technique was judged to be unsafe, and two patients had incomplete resection of the cancer (Fig. 1). The StO2 and T-HbI values were successfully acquired without problems and analyzed intraoperatively in a real-time manner in all 43 included patients. There were no intraoperative incidents during esophagectomy. The patients’ demographic characteristics are shown in Table 1. The cohort undergoing esophageal cancer surgery had no unusual characteristics in terms of average patient age, sex, body size, or anesthesia risk. Table 2 shows the operative data. Most esophagectomies were performed using the thoracoscopic and laparoscopic approaches. All patients underwent three-field lymph node dissection and received a narrow gastric conduit. The most frequent reconstruction route was retrosternal. Mechanical anastomosis using the modified Collard technique was performed in all 43 patients.

**Table 1:** Patients’ demographic characteristics

Variable	Values (*n* = 43)
**Age (mean ± S.D.)**	67.5 ± 7.4
**Gender (M**: **F)**	36: 7
**Body Mass Index** **(mean ± S.D.)**	21.7 ± 1.9
**ASA Grade**	
**Grade 1**	22
**Grade 2**	21
**Basic Disease**	
**Cerebrovascular**	2
**Cardiovascular**	7
**Respiratory**	10
**Diabetes**	9
**Main location of tumor**	
**Upper**	8
**Middle**	19
**Lower**	16
**Preoperative treatment**	
**Neoadjuvant chemotherapy**	21
**No neoadjuvant chemotherapy**	22
**Clinical stage (UICC 7th)**	
**Stage I**	11
**Stage II**	5
**Stage III**	23
**Stage IV**	4

**Table 2:** Patients’ operative data

Variables	Values (*n* = 43)
**Total time of surgery (mean ± S.D.)**	438.4 ± 45.7
**Total amount of blood loss (mean ± S.D.)**	115.6 ± 107.6
**Type of thoracic approach**	
**Thoracoscopic**	42
**Thoracotomy**	1
**Type of abdominal approach**	
**Laparoscopic**	40
**Laparotomy**	3
**Field of lymph dissection**	
**Two fields**	2
**Three fields**	41
**Route of reconstruction**	
**Retrosternal**	39
**Posterior mediastinal**	4
**Type of anastomotic procedure**	
**Mechanical liner**	43
**Others**	0

**Fig. 5:**
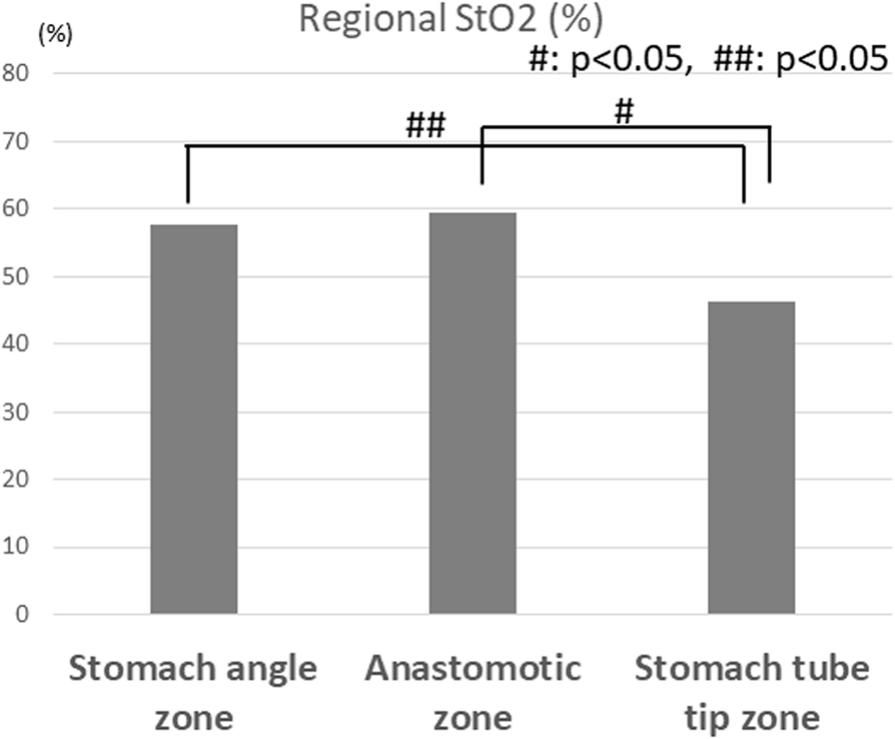
Mean value of tissue oxygen saturation at three zones of the gastric tube. Tissue oxygen saturation gradually decreased from the caudal side to the cranial side. No statistically significant differences in tissue oxygen saturation were found between the stomach zone and the anastomotic zone. However, there was a significant decrease in tissue oxygen saturation in the stomach tube tip zone compared to the anastomotic zone. These results suggest that oxygen saturation is maintained to some extent by intramural blood flow in the stomach in the gastroepiploic region beyond the end of the right gastroepiploic artery, but that it is difficult to maintain oxygenation beyond the demarcation line, resulting in a decrease in oxygen saturation

A representative video of an intraoperative evaluation of the gastric tube is shown in Supplemental Video 1. The regional StO2 values for each region of interest on the reconstructed organ, namely the stomach tube, are shown in Fig. 5. The StO2 was generally well preserved in the stomach angle zone that was supplied by the right gastroepiploic artery (Fig. 4). The StO2 values in the anastomotic zone was sufficient after the direct right gastroepiploic arterial blood supply was lost, and did not differ from the StO2 values in the stomach angle zone. However, the StO2 decreased markedly around the tip of the gastric tube in the area beyond the demarcation line (Figs. 4, 5).

**Fig. 6:**
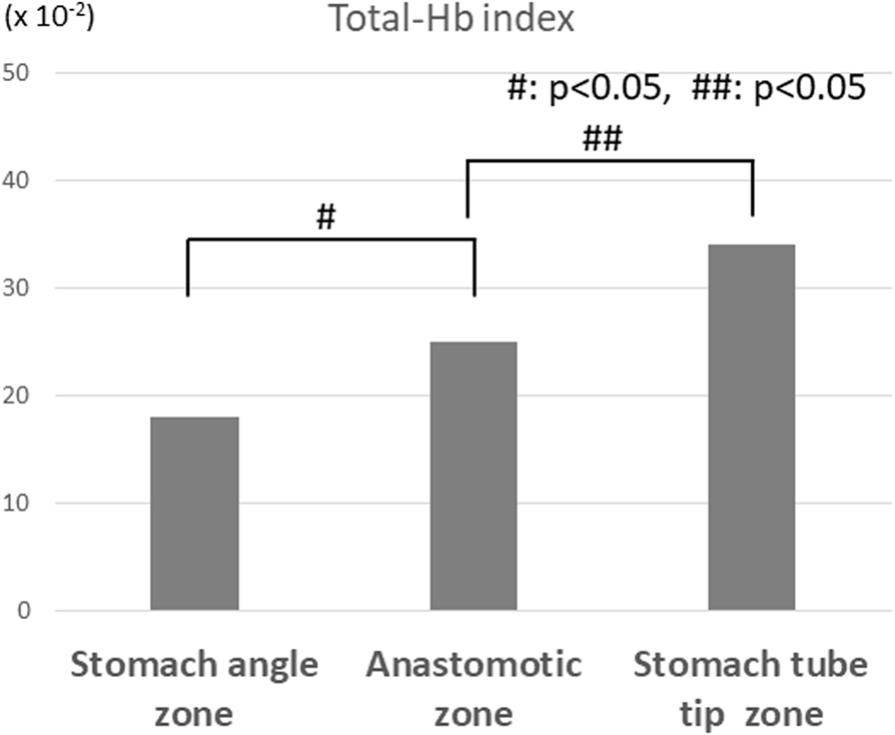
Mean value of T-Hb index at three zones of the gastric tube. T-Hb index values gradually increased from the caudal side to the cranial side. Statistically significant differences in T-Hb index values were found between the stomach angle zone and the anastomotic zone (*p* < 0.05). A statistically significant difference in T-Hb index was also observed between the anastomotic zone and the stomach tube tip zone (*p* < 0.05). These results indicate that the progression of congestion toward the tip of the gastrointestinal tract is gradual and objective

Details of the StO2 and T-HbI within the anastomotic zone, the most important region, are given below. Figure 6 shows the comparison of the mean T-HbI values at each site of the gastric tube. In principle, the T-HbI is an indicator of tissue congestion. The T-HbI value was lowest at the stomach angle zone, then gradually significantly increased toward the stomach tube tip zone.

Postoperative outcomes are shown in Table 3. There were no in-hospital deaths and 86.0% of patients successfully accomplished the CMP. The median postoperative hospital stay was 16 days. The incidences of anastomotic leakage, anastomotic stricture, postoperative pneumonia, and postoperative vocal cord paralysis were 6.9.%, 9.3, 16.2, and 25.5%, respectively.

**Table 3:** Postoperative outcomes

Variables	Values (*n* = 43)
**Anastomotic leakage (%)**	3 (6.9%)
**Anastomotic stricture (%)**	4 (9.3%)
**Pneumonia (%)**	7 (16.2%)
**Vocal cord paralysis (%)**	11 (25.5%)
**Success accomplishment of CMP (%)**	37 (86.0%)
**In-hospital death (%)**	0 (0%)
**Postoperative hospital stays (range)**	16 (12–88)

*CMP* clinical management pathway

**Fig. 7:**
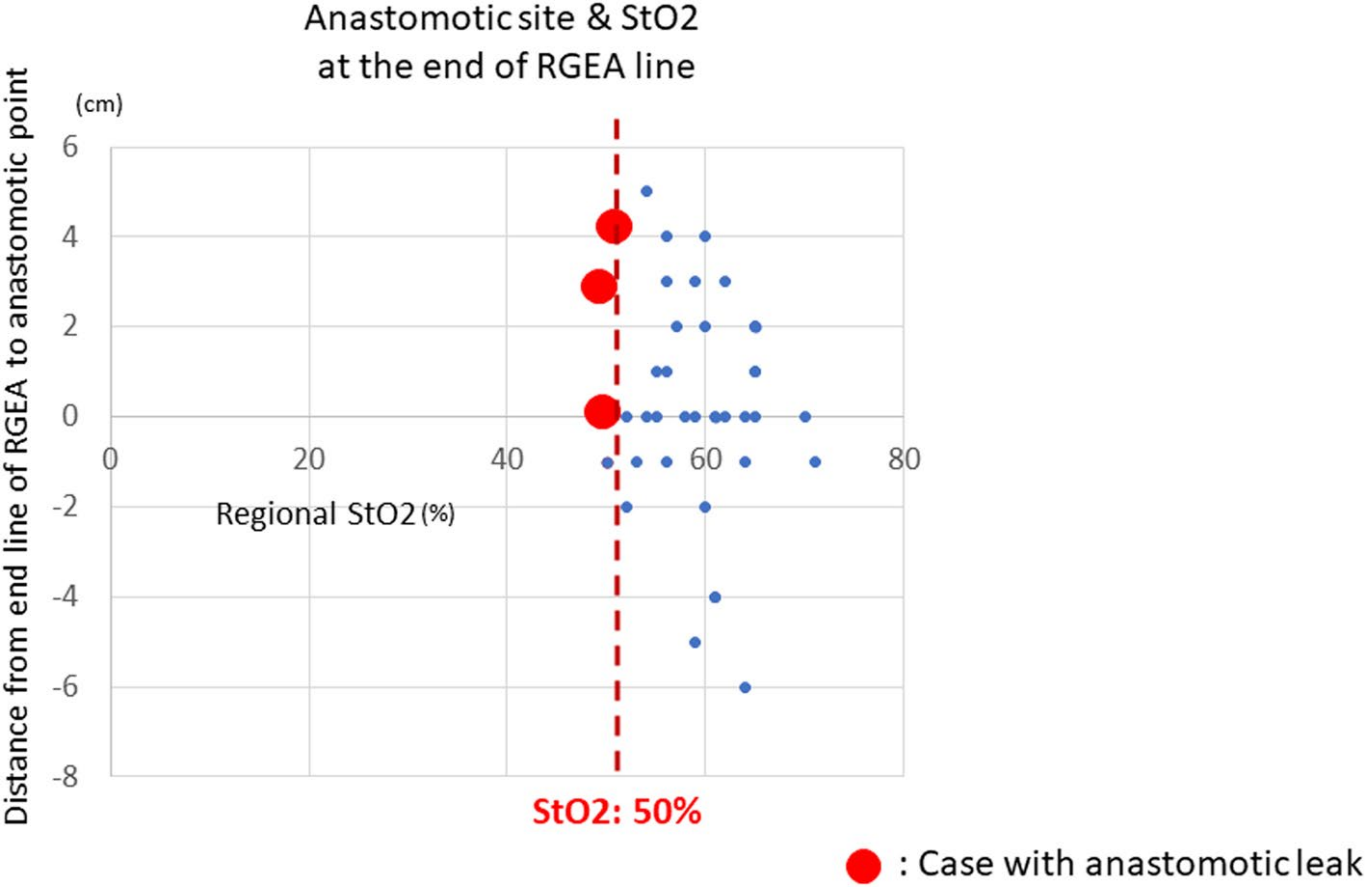
Relationship between anastomotic site and value of tissue oxygen saturation of the stomach tube. The actual anastomotic position in the final gastric tube and the distribution of tissue oxygen saturation (%) at the terminal line of the RGEA are shown as oral (+) and anal (−) from the terminal line of the RGEA. Tissue oxygen saturation generally ranged from 50 to 70% in most cases. The actual anastomotic position varied. In cases with anastomotic leakage, the anastomotic position was on the oral side of the terminal line of the RGEA. Tissue oxygen saturation in the anastomotic leakage cases was below 50% in all cases. (Cases with suture failure are indicated by red dots)

**Fig. 8:**
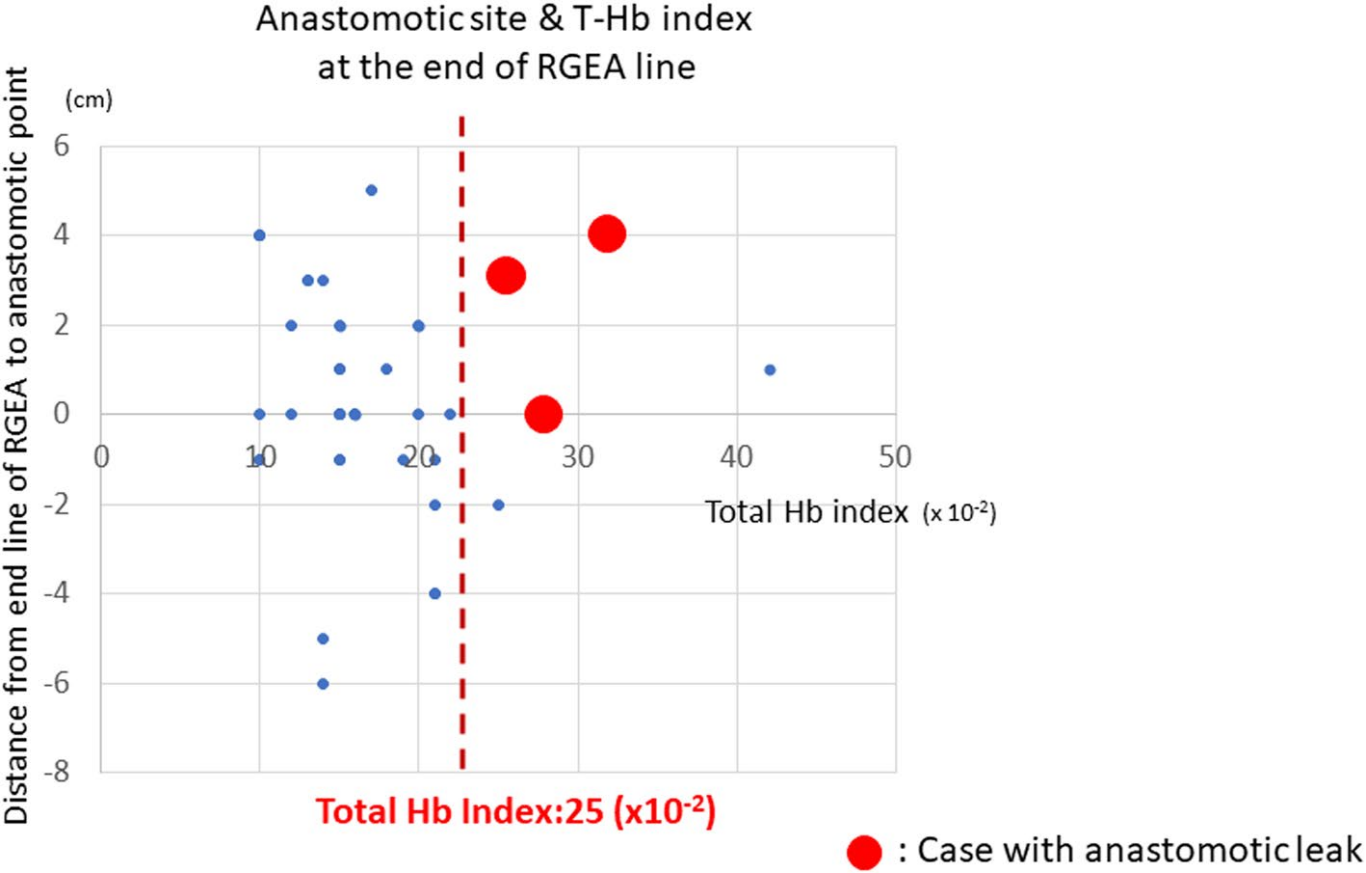
Relationship between anastomotic site and value of T-Hb index of the stomach tube. The actual anastomotic position of the final gastrointestinal tube and the distribution of T-Hb index values at the terminal line of RGEA are shown as oral (+) and anal (−). The T-Hb index values were generally divided by 10–25 (× 10^−2^) in most cases. The actual anastomotic positions were varied. The T-Hb index values of anastomotic leakage cases were all higher than 25 (× 10^−2^). (Cases with suture failure are indicated by red dots)

During the reconstruction process in esophagectomy, the line of anastomosis was selected based on the blood perfusion as determined by the StO2 and T-HbI as well as tension considerations. Most anastomosis sites were within 3 cm of the end of the right gastroepiploic artery. The relationships between the distance from the site of anastomosis to the end of the right gastroepiploic artery and the StO2 and T-HbI values for each patient are shown in Figs. 7 and 8.

Figure 7 shows the relationship between the location of the actual anastomosis site of the stomach tube and the StO2 value in the 43 patients. In most cases, anastomosis was performed at plus or minus 3 cm of the end of the right gastroepiploic artery line, and most cases had the anastomosis performed at a position more cephalic than the line at the end of the right gastroepiploic artery. Anastomotic leakage was not observed in cases in which the StO2 value at the right gastroepiploic artery line remained above 50%, even if the anastomosis was performed at the tip of the gastric tube. In contrast, the StO2 value at the right gastroepiploic artery line was less than 50% in all three patients with anastomotic leakage.

Figure 8 shows the relationship between the location of the actual anastomosis of the stomach tube and the T-HbI values of the 43 patients. Anastomotic leakage was not observed in most patients who were able to maintain a congestive state with a T-HbI value at the line at the end of the right gastroepiploic artery of 25 × 10^−2^ or less, even if the anastomosis was performed at the tip of the stomach tube. In contrast, the T-HbI value at the end of the right gastroepiploic artery was higher than 25 × 10^−2^ in all three patients with anastomotic leakage.

Table 4 shows the mean StO2 values at the line of the end of the right gastroepiploic artery, demarcation line, and anastomosis site in patients grouped according to the presence or absence of postoperative anastomotic leakage. The mean StO2 was significantly lower in the group without anastomotic leakage versus the group with leakage at the line at the end of the right gastroepiploic artery (*P* < 0.01), demarcation line (*P* < 0.01), and anastomosis site (*P* < 0.01). Moreover, the mean T-HbI values were significantly lower in the group without anastomotic leakage than the group with leakage at the line at the end of the right gastroepiploic artery (*P* < 0.01), demarcation line (*P* < 0.01), and anastomosis site *(P* < 0.01) (Table 5).

**Table 4:** Mean tissue oxygen saturation at two gastric tube points and the point of anastomosis in patients who did and did not experience postoperative anastomotic leakage

Variables	Mean StO2 (%) No-leakage group (*n* = 40)	Mean StO2 (%) leakage group (*n* = 3)	*p*-value
**Demarcation line (%)**	59.1 ± 1.9	46.3 ± 2.2	< 0.01
**End of RGEA line (%)**	58.6 ± 2.1	48.9 ± 2.6	< 0.01
**Anastomotic line (%)**	58.8 ± 3.1	48.3 ± 2.4	< 0.01

Values are means ± standard deviation

*RGEA* Right gastroepiploic artery

**Table 5:** Mean tissue total Hb index at two gastric tube points and the point of anastomosis in patients who did and did not experience postoperative anastomotic leakage

Variables	Mean Hb index No-leakage group (*n* = 40)	Mean Hb index leakage group (*n* = 3)	*p*-value
**Demarcation line (× 10**^**−2**^**)**	23.7 ± 7.2	37.2 ± 1.9	0.01
**End of RGEA line (× 10**^**−2**^**)**	18.6 ± 4.1	28..1 ± 2.0	0.01
**Anastomotic line (× 10**^**−2**^**)**	20.1 ± 5.8	28.5 ± 3.3	0.01

Values are means ± standard deviation

*RGEA* Right gastroepiploic artery

Supplemental Table 1 shows the StO2 and T-HbI values at the two height lines of the gastric tube and the height line of the anastomosis. The regional StO2 significantly decreased from the end of the right gastroepiploic artery through the anastomotic zone to the demarcation line *(P <* 0.01). The mean T-HbI significantly increased from the end of the right gastroepiploic artery through the anastomotic zone to the demarcation line (*P* < 0.01); even within the anastomotic zone, there was a clear increase in congestion toward the tip of the stomach tube.

## Discussion

Anastomotic leakage after esophageal cancer surgery has a significant impact on not only the short-term outcomes but also the medium-term quality of life [12, 13]. It has recently been reported that perioperative anastomotic leakage also affects the long-term oncologic prognosis [14, 15].

Failure of the anastomosis is caused by a variety of risk factors. Among these multiple risk factors, some of the most important are the preoperative nutritional status and presence of pre-existing metabolic diseases, as well as the pre- and perioperative management strategies, such as preoperative rehabilitation [16]. Another important patient-specific factor is the presence of a small stomach, which makes it difficult to create a long enough gastric tube [17]. However, one of the most direct and important factors affecting anastomotic failure are the blood flow status of the stomach tube and the determination of its optimal anastomotic site [18, 19].

The ICG method is currently the most widely used method for assessing the blood flow status of the stomach tube and determining the optimal site of anastomosis. A recent systematic review has shown that the use of the ICG technique in combination with the ICG method contributes to a reduction in the incidence of anastomotic leakage [20], and there is unanimous support for its usefulness among surgeons. However, the ICG method is a visually intuitive evaluation method that is difficult to evaluate objectively [21]. The ICG method has several inadequacies, including: 1) various biases in evaluation, 2) difficulty in establishing objective indices, 3) difficulty in evaluating the congestive status although the inflowing blood flow can be measured, 4) difficulty in performing and reevaluating the method repeatedly, and 5) the need for caution in patients with a history of contrast agent allergy [22, 23]. In particular, measurement bias has a significant influence on the results, and it is conventionally noted that although a closer proximity allows for darker staining, a few cm difference in the distance between the camera and the target organ can significantly change the results [24].

The novel device used in the present study may eliminate the various shortcomings of the ICG method listed above. The device provides numerical values as objective indicators and enables repeated measurements. In addition, ischemia can be measured based on the StO2, and the congestive state can be simultaneously evaluated in real time based on the T-HbI. Furthermore, there are no safety concerns related to the injection of a reagent into the body. This novel device has the potential to evaluate organ blood flow not only in other types of gastrointestinal cancer surgery but also in certain types of reconstructive plastic surgery. Evaluation of the stomach tube using this device showed that the StO2 was maintained even at the tip of the stomach tube, rather than at the site receiving direct blood flow from the right gastroepiploic artery, and that the StO2 was markedly decreased distal to the demarcation boundary line. The present results show that if the StO2 is above 50% and the T-HbI is below 25 × 10^−2^, this site may be judged as safe for the anastomotic position.

Based on the results of this study, if the gastric tube can only be raised to a position that does not meet the criteria of StO2 of 50% or more and T-HbI of 25 or less, the following measures can be assumed, although anastomotic leakage does not always occur.

First, in order to further secure the elevation, Kocher’s duodenum mobilization and detachment of adhesions at the base of the stomach tube should be performed to the maximum extent possible, and efforts should be made to increase the elevation as close as possible to the optimal target anastomotic site. Second, to minimize the risk of anastomotic leakage, additional measures such as omentoplasty and the use of an automatic anastomosis device with reinforcement should be added. Third, even in the event of anastomotic leakage, remnant omental tissue filling of the dead space and positioning of the drain to minimize the risk of serious condition.

The present study has several limitations. First, it was a prospective study conducted in a single institution. A multicenter study is required to show the efficacy of this novel device for analyzing the StO2 and T-HbI, and demonstrate its superiority over ICG imaging. Second, the thoracic and abdominal surgical devices slightly changed over the course of the study period. However, this change was caused by the introduction of the robotic approach, while the anesthesia and perioperative patient management remained consistent, which is a strength of the study. Third, the surgical approaches were not uniformly fixed; several patients underwent laparotomy because they had a history of laparotomy or had bulky lymph node metastases in the abdominal area. However, the incidence of anastomotic leakage did not differ in accordance with the anastomosis procedure.

## Conclusions

In conclusion, the novel device that measures the regional StO2 and T-HbI provides real-time intraoperative measurements of the StO2 and supposed tissue congestion level and is useful in determining the optimal site for anastomosis to minimize the risk of anastomotic leakage in patients undergoing thoracic esophagectomy for esophageal cancer. Future large-scale randomized controlled studies are warranted to confirm our findings and demonstrate the superiority of this novel device over ICG imaging.

### Supplementary Information

**Additional file 1: Supplemental Video1.** Video of real time intraoperative evaluation of tissue oxygen saturation and T-Hb index during surgery. Tissue oxygen saturation and T-Hb index are performed after gastric tube preparation. A sterile bag is used to cover this device, and the optimal anastomotic site is measured in real time.

**Additional file 2: Supplemental Table 1.** Tissue oxygen saturation values at two points of the gastric tube and the point of anastomosis

## Data Availability

The datasets used and/or analysis during the current study are available from the corresponding author on reasonable request.

## Abbreviations

StO2: Tissue oxygen saturation
T-HbI: Total hemoglobin index
LED: Light emitting diode
RGEA: Right gastroepiploic artery (RGEA)
ICG: Indocyanine green
