# Supplementary material for: A novel device to assess the oxygen saturation and congestion status of the gastric conduit in thoracic esophagectomy
Source: BMC Surg. 2024 Jan 8;24:17. doi: 10.1186/s12893-023-02303-0 (PMC10775575; doi:10.1186/s12893-023-02303-0)
Supplement: Supplementary file 2 — Additional file 2: Supplemental Table 1. Tissue oxygen saturation values at two points of the gastric tube and the point of anastomosis [file 12893_2023_2303_MOESM2_ESM.docx]

**Supplemental Table 1** Tissue oxygen saturation values at two points of the gastric tube and the point of anastomosis

| **Variables** | **Hight line of the Stomach tube** | | |  |
| --- | --- | --- | --- | --- |
|  | **End of RGEA line** | **Anastomotic line** | **Demarcation**  **line** | **p- value** |
| **Mean values of**  **Regional StO2 (%)** | 58.8 ± 2.2 | 57.3 ± 3.5 | 55.4 ± 2.7 | P < 0.01 |
| **Mean values of**  **Total Hb Index (x10^-2^)** | 17.8 ± 1.9 | 21.6 ± 3.0 | 23.7 ± 3.8 | P < 0.01 |

RGEA: Right gastroepiploic artery
